# The Role of Long-Term DNA Replication Stress Induced by Hydroxyurea in Meristematic Root Cells of *Allium cepa*

**DOI:** 10.3390/ijms27219995

**Published:** 2026-11-07

**Authors:** Aneta Żabka, Natalia Gocek-Szczurtek, Mateusz Wróblewski, Klaudia Cieślak, Justyna Teresa Polit

**Affiliations:** 1 Department of Cytophysiology, Faculty of Biology and Environmental Protection, University of Lodz, 90-236 Łódź, Poland; natalia.gocek@edu.uni.lodz.pl (N.G.-S.); mateusz.wroblewski2@edu.uni.lodz.pl (M.W.); justyna.polit@biol.uni.lodz.pl (J.T.P.); 2 Department of Pathomorphology, Synevo, 90-236 Łódź, Poland; klaudia.labuk@synevo.pl

**Keywords:** *Allium cepa*, DNA replication stress, H3K56Ac, hydroxyurea, oxidative stress

## Abstract

Replication stress (RS) is a process characterized by impaired DNA replication, resulting in the slowing down or arrest of replication forks (RFs) and in the activation of the DNA damage response. The aim of this study was to investigate the effects of 24, 48, 72 and 96 h exposures of *Allium cepa* root meristematic cells to hydroxyurea (HU) at concentrations of 1, 2, 3 and 4 mM on cell nucleus morphology, DNA replication dynamics, mitotic activity and the production of reactive oxygen species (ROS), including hydrogen peroxide (H_2_O_2_) and superoxide anion (O_2_∙^−^). Furthermore, to assess epigenetic changes associated with DNA damage, the level of acetylation of lysine 56 of histone H3 (H3K56Ac) was analyzed. It was demonstrated that incubation of roots in 1 mM HU leads to the appearance of so-called interphase–mitotic (I-M) cells. Furthermore, as the HU concentration increased, inhibition of mitotic activity, a slowing down or partial inhibition of DNA replication, and the accumulation of reactive oxygen species (ROS) were observed. Furthermore, after 24 and 48 h of incubation with 1, 2 and 3 mM HU, a similar increase in the mean fluorescence intensity of H3K56Ac was observed, with the highest intensity recorded following treatment with 4 mM HU. Prolongation of the incubation time with the inhibitor to 72 and 96 h led to a further increase in fluorescence in the cell nuclei. The obtained results indicate that the increase in H3K56Ac levels may be associated with the accumulation of DNA damage under conditions of prolonged exposure to HU.

## 1. Introduction

In eukaryotic cells, DNA replication is a strictly regulated process that ensures the faithful duplication of genetic material. Bidirectional replication forks (RFs) are initiated at numerous replication origins, which must obtain a so-called ‘licence’ before the start of the S-phase. This capability is achieved through the binding of replication-initiating proteins, which prepare the chromatin structure for DNA synthesis [1,2]. Disruptions in the course of DNA replication can lead to the formation of stalled RFs, but the cell possesses S-phase-specific mechanisms that enable it to cope with such disorders. If RFs become stalled (e.g., due to DNA damage, nucleotide deficiency or structural obstacles in chromatin), they can be restored by RF restart processes [3]. However, if stalled RFs cannot be restarted or rescued, they undergo irreversible inactivation, known as ‘replication fork collapse’. In such situations, double-strand breaks (DSBs) in DNA often occur, leading to the activation of DNA damage response (DDR) pathways [2]. This mechanism involves cell cycle arrest in response to environmental changes [4]. In the case of minor DNA damage, cell cycle checkpoints are activated, leading to a temporary arrest of the cell cycle, which allows stimulation of repair processes before division resumes [5,6]. To initiate cell cycle arrest, the Ser/Thr kinases ATM and ATR must be activated; these are triggered in response to DNA damage: ATR responds primarily to single-strand breaks (SSBs), while ATM responds to double-strand breaks (DSBs) [4]. Subsequently, ATM and/or ATR phosphorylate the transcription factor SOG1 (Suppressor of Gamma Response 1), which is the functional homologue of p53 in plants [7]. The activated SOG1 protein then binds to the promoter regions of target genes. These (among others) include genes involved in DNA repair, genes that initiate programmed cell death (PCD), and genes responsible for cell cycle arrest [7,8].

Although the mechanism of DNA replication is characterized by high accuracy, it involves numerous challenges and its accuracy can be disrupted by various stress factors [9,10]. This phenomenon, generally referred to as replication stress (RS), is a significant source of genomic instability, which exposes cells to mutagenesis and/or death [11]. Physical barriers limiting DNA replication include both endogenous obstacles, such as secondary DNA structures (e.g., hairpin or G-quadruplex structures) and chromatin-associated proteins, as well as exogenous factors, e.g., genotoxins. The latter include reactive intermediates of cellular metabolism, environmental chemicals and ultraviolet (UV) radiation [9,10]. Depletion of the deoxyribonucleoside triphosphate (dNTP) pool also constitutes a significant source of RS, leading to a slowdown or arrest of DNA polymerase activity during the elongation process; for this reason, dNTP levels are strictly regulated during the S-phase of the cell cycle [10,12,13,14].

Hydroxyurea (HU) induces RS by inhibiting ribonucleotide reductase (RNR) activity, thus leading to a depletion of the dNTP pool and, consequently, to the inhibition of DNA biosynthesis [15]. Using the root apical meristems of *Allium cepa*, an excellent model for cytological studies characterized by a high DNA content (33.4 pg) and large chromosomes, our current study aims to determine whether, and to what extent, the prolonged 24, 48, 72 and 96 h treatments with 1, 2, 3 and 4 mM HU results in the arrest of the replicative capacity and mitotic activity of the cells. Since previous studies have shown that 72 h incubations of *A. cepa* [16,17,18,19,20] and *Vicia faba* [21] root meristems in a 0.75 mM HU solution lead to asynchronous nuclear chromatin condensation, i.e., the appearance of I-M (interphase–mitotic) cells, we investigated whether higher HU concentrations may also induce the appearance of atypical cells characterized with intranuclear asynchrony. Furthermore, the extent of the cells’ response to RS have been tested at both cytophysiological and molecular levels. To achieve this, root meristem cells of *A. cepa* subjected to prolonged treatment with 1, 2, 3 and 4 mM HU were examined for nuclear and chromosomal morphology, intranuclear production of hydrogen peroxide (H_2_O_2_), using 3,3-diaminobenzidine (DAB) staining, and superoxide anion (O_2_∙^−^), using nitroblue tetrazolium (NBT) staining. To assess HU-induced epigenetic changes associated with the activation of the DNA damage response under RS conditions [22,23,24,25], an analysis of the acetylation level of histone H3 at lysine 56 (H3K56Ac) was carried out using immunocytochemistry.

## 2. Results

### 2.1. Prolonged RS Creates Biphasic Interphase–Mitotic (I-M) Cell Nuclei

**Figure 1:**
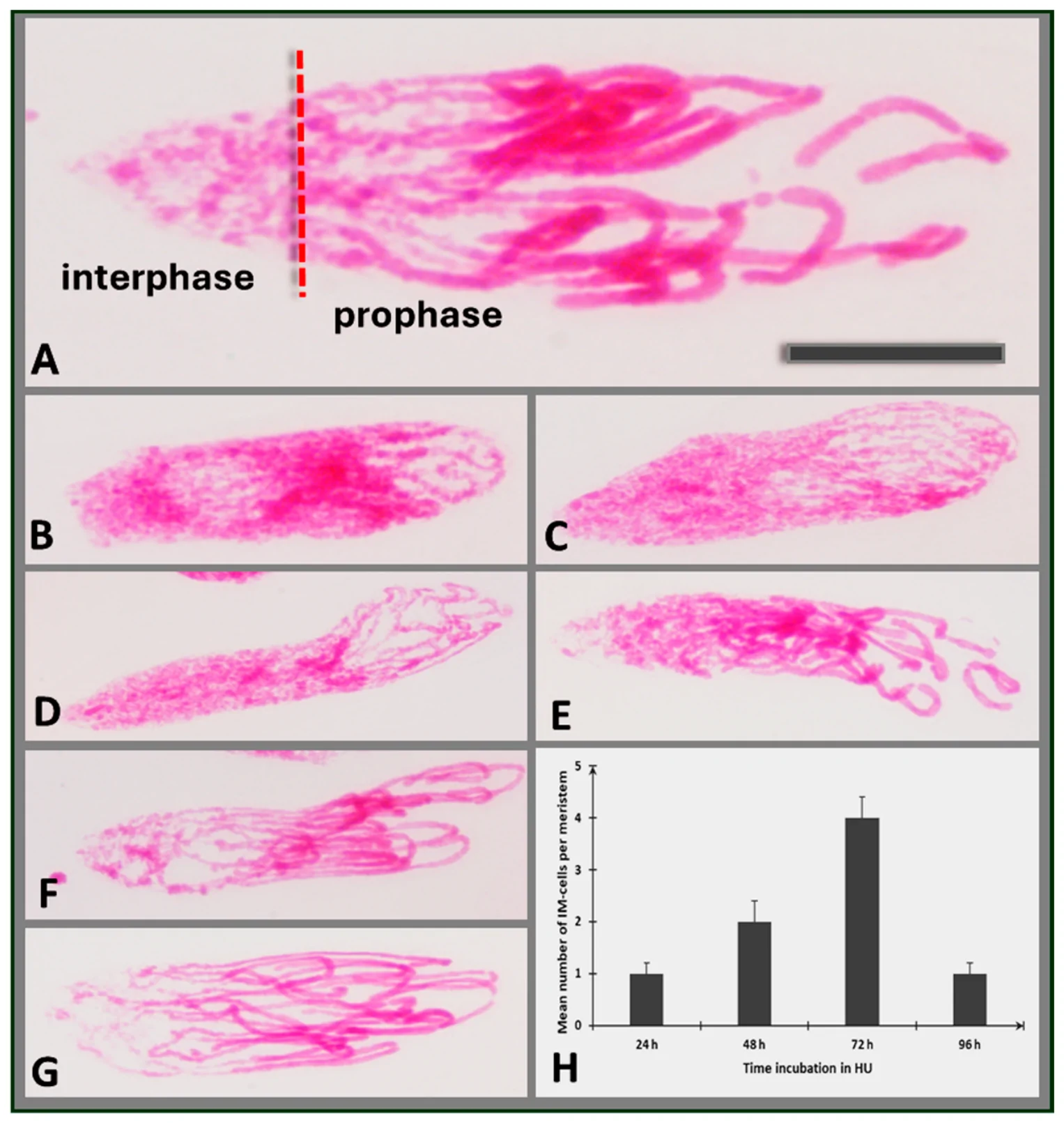
Biphasic interphase–mitotic (I–M) nuclear organization in *A. cepa* root meristem cells following treatment with 1 mM HU. (**A**–**G**) Representative nuclei showing the coexistence of decondensed and partially condensed chromatin. Scale bar = 10 μm. (**H**) The mean number of I-M cells per meristem after 24, 48, 72 and 96 h incubation in 1 mM HU. Error bars represent standard deviation (SD); *n* = 3 biological replicates; approximately 100 nuclei were analyzed per treatment.

Continuous incubation oI-M*A. cepa* root meristems with 1 mM HU results in a fraction of cells showing biphasic nuclear organization of the interphase–mitotic type (I-M; Figure 1A–G). These structures were not observed after treatment with higher concentrations of HU (2, 3, and 4 mM). The morphology of I-M–M nuclei was characterized by the co-existence, within a single nucleus, of relatively decondensed chromatin typical of interphase and partially condensed chromatin resembling the prophase (Figure 1A–G). Furthermore, the mean number of IM cells per meristem was recorded for all periods of incubation in the inhibitor (Figure 1H). Results of this analysis clearly indicate the rarity of the formation of biphasic I-M structures; after the 72 h treatment with 1 mM HU, the mean number of IM cells per meristem reached a value of approximately 4 (Figure 1H).

**Figure 2:**
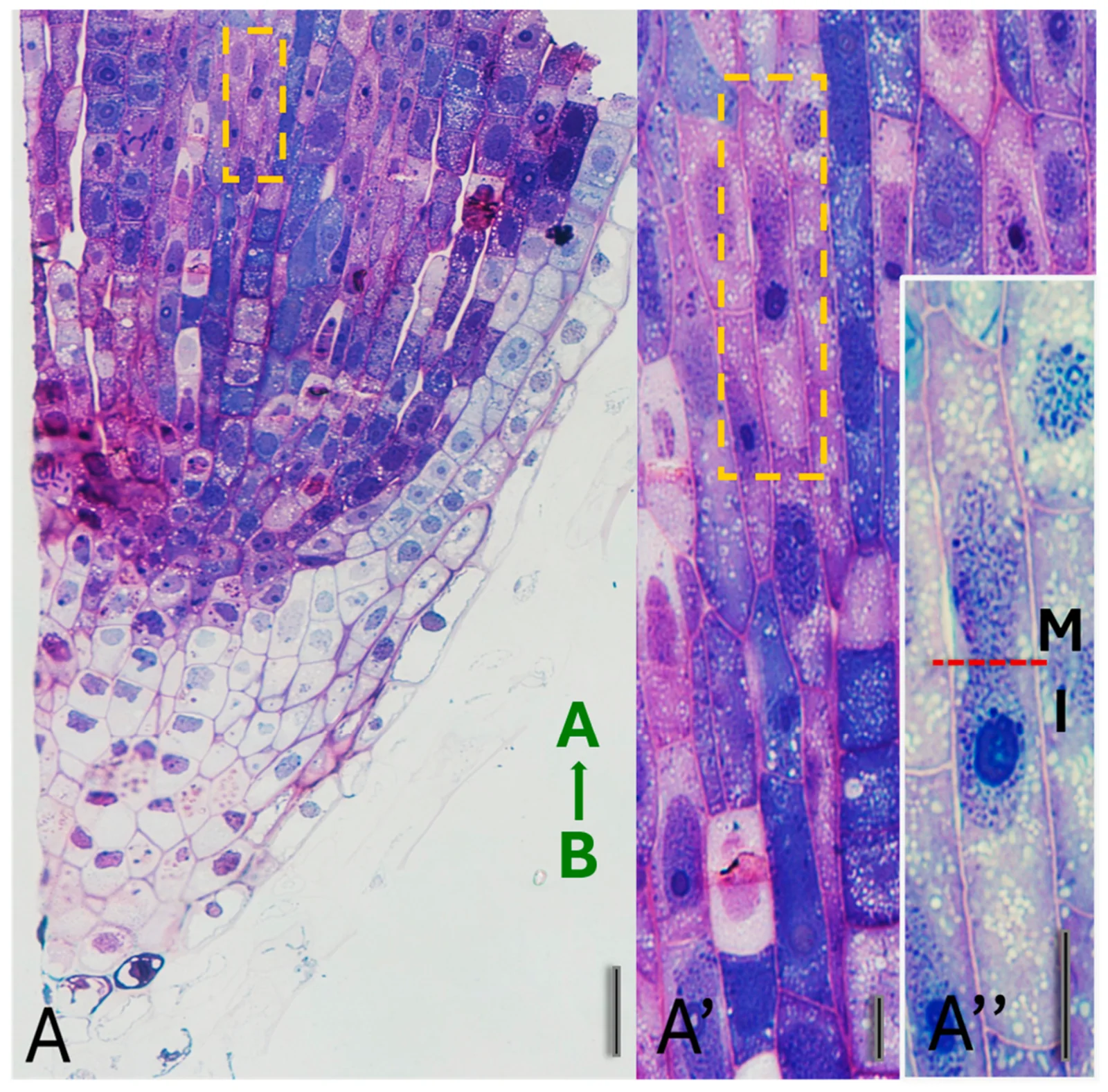
The effect of 72 h incubation with 1 mM HU on meristematic root cells of *A. cepa*. (**A**) Longitudinal section of the apical part of the root with basipetal-to-acropetal orientation indicated as B → A and localization site of the I-M cell (surrounded by closed dotted lines). Scale bar = 50 μm. (**A′**,**A″**) The same I-M cell at higher magnification. Scale bars = 10 μm.

Consistent with our previous observations [19], no relationship was found between the acropetal orientation of the root axis (basis to apex polarity) and the direction of chromatin condensation (manifested by nuclear I or M poles facing the root apex). Microscopic examination of 100 semi-thin sections from the root meristems of *A. cepa*, following 72 h of treatment with 1 mM HU, revealed an almost perfect balance between the frequency of occurrence of biphasic I-M nuclei whose I or M poles are oriented in line with the root’s basis-apex direction (Figure 2A,A′,A″). It was shown that approximately 48 per cent of nuclear I poles were directed towards the root apex, while the remaining 52 per cent consisted of M poles directed towards the apex.

### 2.2. Changes in Mitotic Activity Induced by RS

**Figure 3:**
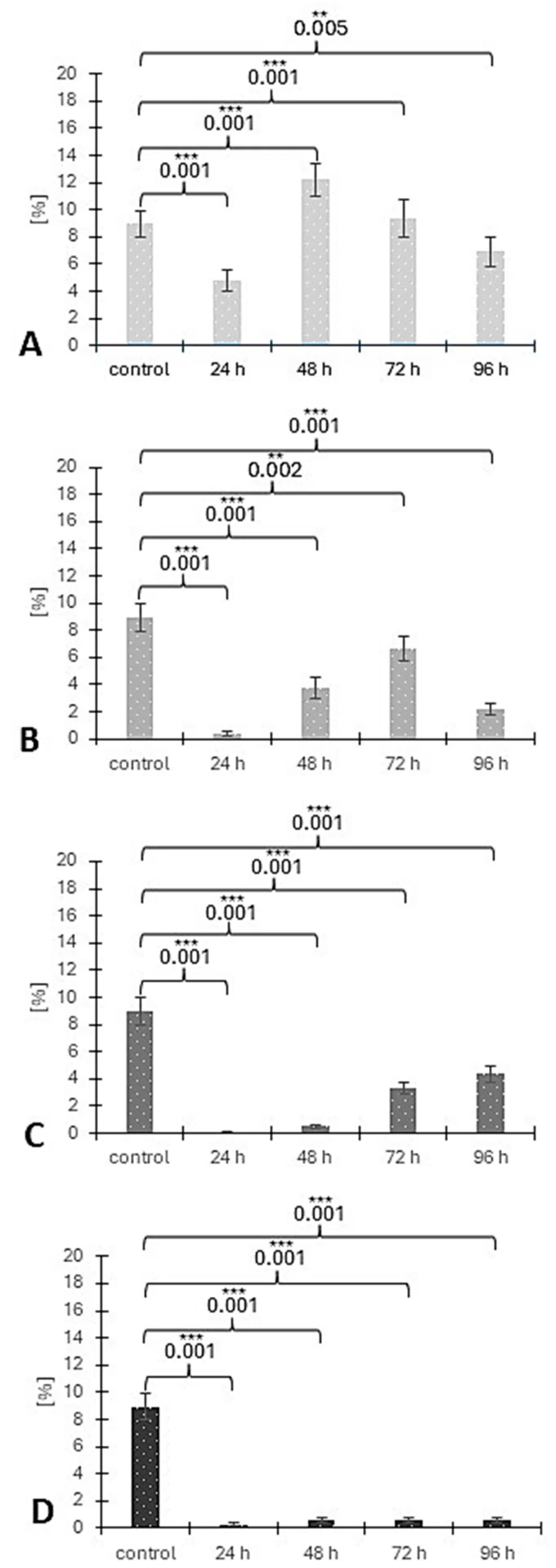
Mitotic indices (% ± S.D.) in onion root meristem cells after 24, 48, 72 and 96 h incubations with: (**A**) 1, (**B**) 2, (**C**) 3 and (**D**) 4 mM HU. Mean values of the mitotic index were calculated based on five root meristems, with 200 cells analyzed per meristem (*n* = 3 biological replicates). When compared with the control, statistically significant changes in mean values (±SD) are marked by asterisk: *** *p* < 0.001, ** *p* < 0.005, and ** *p* < 0.002 (Student’s *t*-test).

As shown in Figure 3, incubation of onion root meristems in the presence of the lowest HU concentration (1 mM) resulted in a reduction of approximately 50% in the relative number of mitotic cells, compared with the control (Figure 3A). After 48 h of HU treatment, an increase in mitotic activity to approximately 12% was observed. However, at subsequent time points, a gradual decrease in the mitotic index to approximately 7% was recorded (Figure 3A).

The use of HU at a concentration of 2 mM resulted in the inhibition of mitotic divisions as early as in the first day of the treatment (Figure 3B), and the highest mitotic activity was observed after 48 h incubation (approx. 6.6%). In contrast, at the final time point, a decrease in the mitotic index to approximately 2% was recorded (Figure 3B).

As with the 2 mM HU concentration (Figure 3B), 24 h of incubation in the presence of 3 mM HU resulted in complete inhibition of mitotic cell divisions (Figure 3C). The first mitotic cells reappeared 48 h after the start of HU treatment. Over the following days of the experiment, a gradual increase in mitotic activity was observed (Figure 3C).

The most pronounced reduction in mitotic indices was observed at the highest concentration of HU (4 mM). At all time points, the mitotic index remained at a very low level, ranging from 0.2% to 0.6% (Figure 3D).

### 2.3. RS Generates Chromosomal Aberrations

**Figure 4:**
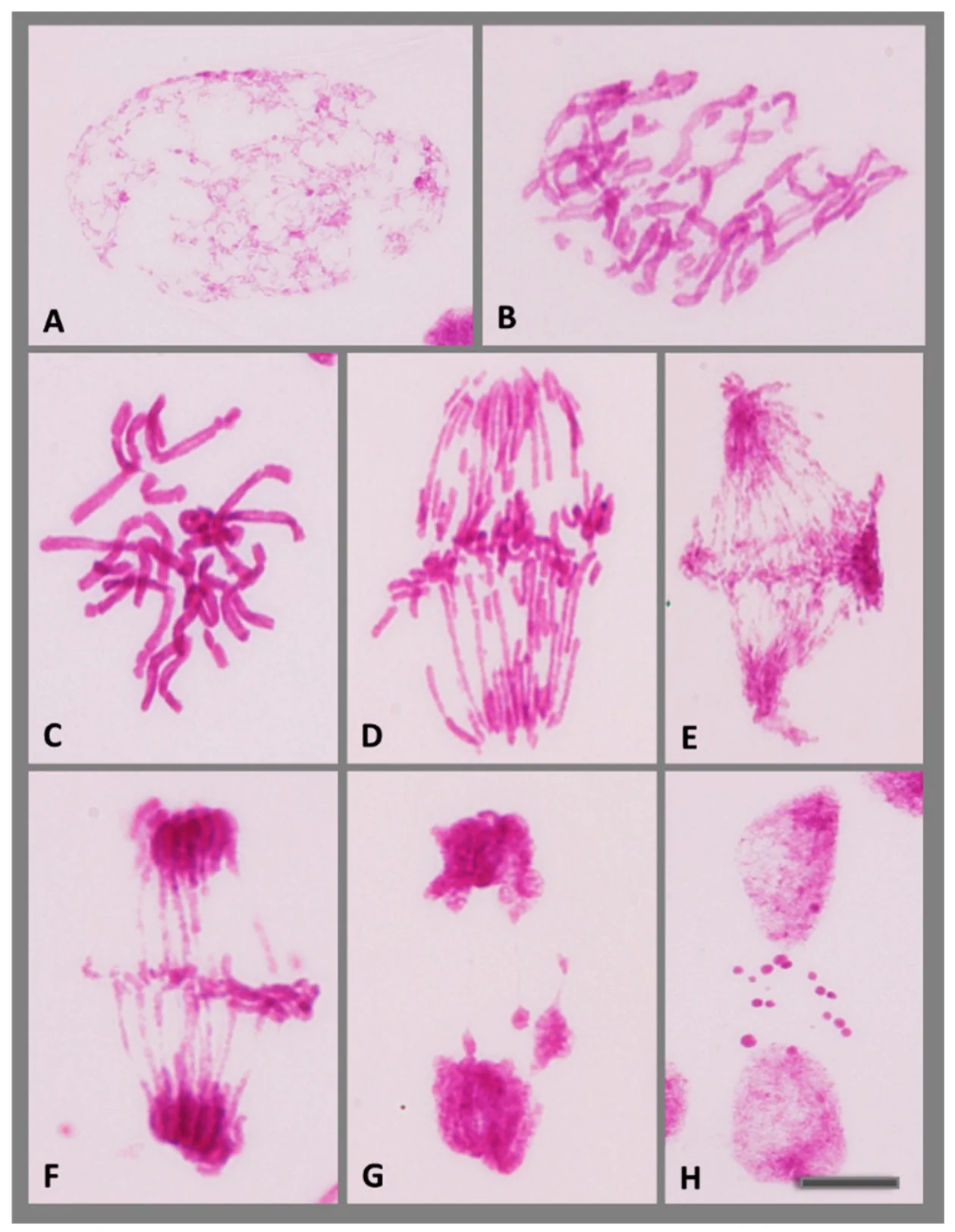
Symptoms of chromosome aberrations after 72 h treatment with 1 mM HU during: (**A**) early prophase, (**B**) late prophase, (**C**) metaphase, (**D**) anaphase), (**E**–**G**) telophase; (**H**) post-telophase cell nuclei with micronuclei. Scale bar = 10 μm.

Microscopic images of Feulgen-stained mitotic cells derived from the root meristems of *A. cepa* treated with 1, 2, 3, and 4 mM HU revealed distinct changes in chromatin architecture. Figure 4A–H show chromosomal aberrations following 24 h treatment with 1 mM HU; similar changes were observed after longer incubation periods and following treatment with 2, 3, and 4 mM HU. It was noted that in both early and late prophase, the chromatin structure exhibited breaks and fractures (Figure 4A,B). In the subsequent mitotic stage, the metaphase, the chromosomes were characterized by a chaotic and disordered arrangement (Figure 4C). In the anaphase and telophase, abnormalities in the normal movement of chromatids towards the opposite poles of the cell were observed. Furthermore, a characteristic feature of RS was the presence of numerous chromosome fragments in the equatorial plane and the formation of chromosome bridges (Figure 4D–F). In telophase and the post-telophase stage, the lost chromosome fragments were ultimately transformed into micronuclei (Figure 4G,H).

**Figure 5:**
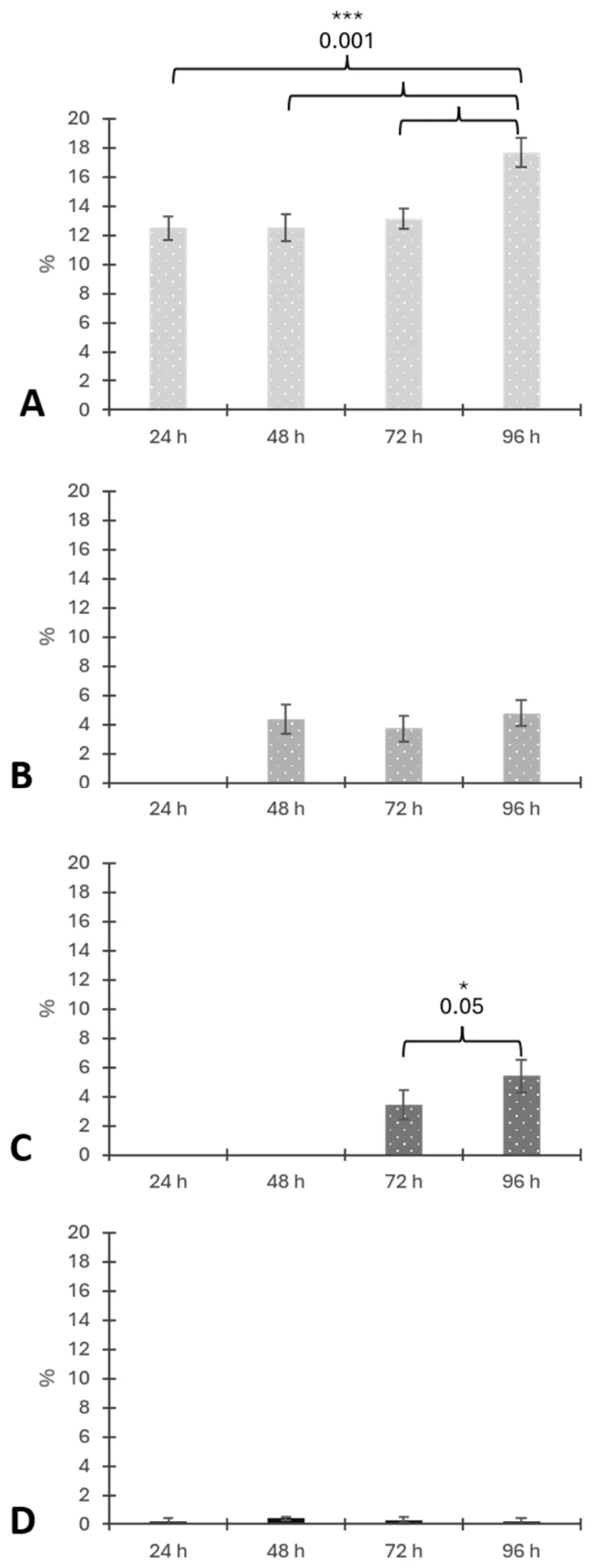
Percentage of aberrant M-phase cells (% ± S.D.) in onion root meristem cells after 24, 48, 72 and 96 h incubations with: (**A**) 1 mM, (**B**) 2 mM, (**C**) 3 mM, and (**D**) 4 mM HU. Mean values of the aberrant index were calculated based on five root meristems, with 200 cells analyzed per meristem (*n* = 3 biological replicates). When compared with the control, statistically significant changes in mean values (±SD) are marked by asterisk: *** *p* < 0.001 and * *p* < 0.05 (Student’s *t*-test).

Incubation of onion root meristems with all concentrations of HU for 24, 48 and 72 h resulted in significant differences in the percentage (%) of aberrant M-phase cells. During the first three days of treatment with 1 mM HU, the relative number of mitotic cells with abnormal chromatin morphology was approximately 12%, and the highest number of abnormalities was recorded after 96 h of exposure (approx. 16%; Figure 5A). Incubation of roots in 2 mM HU resulted in the appearance of cells with an abnormal M-phase only after 48 h of treatment (approx. 4%); after 72 and 96 h, the percentages of aberrant M-phase cells were at a similar level (approx. 3.7% and 4.7%, respectively; Figure 5B). After 3 days of HU treatment, abnormal mitotic cells appeared at 72 (3.5%) and 96 h (4.5%; Figure 5C). In contrast, in *A. cepa* root meristem cells treated with 4 mM HU, due to small number of mitotic cells (Figure 3D), structural abnormalities were observed at a very low level (from 0.2 to 0.4%; Figure 5D).

### 2.4. Analysis of Chromatin Density Profiles

**Figure 6:**
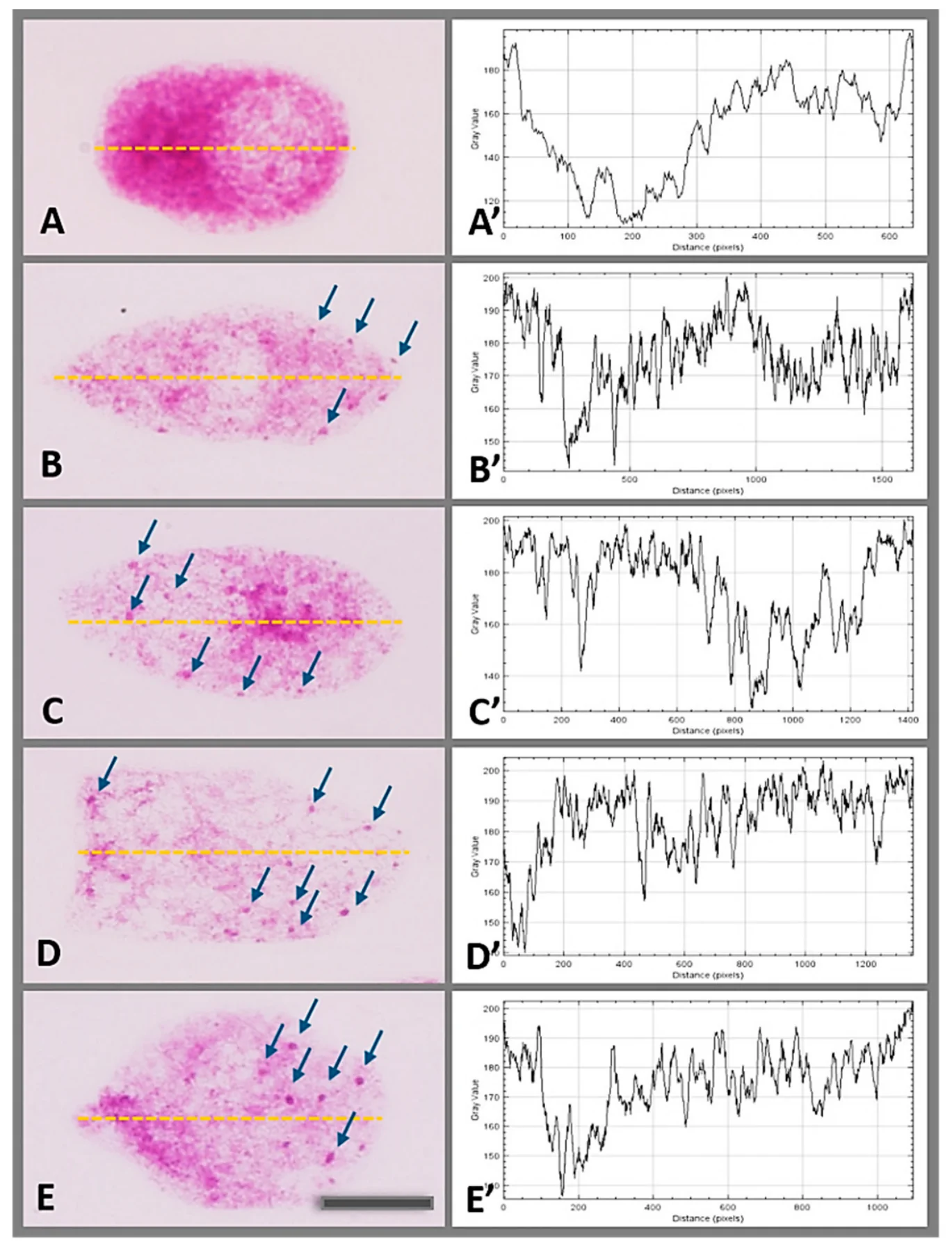
Cell nuclei from meristematic root cells of *A. cepa*: (**A**) control, (**B**) 96 h treatment with 1 mM, (**C**) 2 mM, (**D**) 3 mM and (**E**) 4 mM HU. The chromocentres are indicated by arrows. Scale bar = 10 μm. Corresponding chromatin density profiles: (**A′**) control, (**B′**) 1 mM, (**C′**) 2 mM, (**D′**) 3 mM and (**E′**) 4 mM HU. The dashed line indicates the location of the plot analysis.

Chromatin density profiles were generated using ImageJ software version 1.54p, which enabled the assessment of changes in nuclear chromatin organization following the longest, 96 h exposure to HU at concentrations of 1, 2, 3 and 4 mM (Figure 6). Analysis of the resulting profiles revealed differences compared with the control material (Figure 6A). Regardless of the HU concentration, a more relaxed chromatin structure was observed. At the same time (due to the background effect), areas of increased chromatin density, corresponding to chromocentres, were more clearly defined (Figure 6B–E). These changes suggest that exposure to HU leads to a reorganization of chromatin architecture, involving an increase in contrast between heterochromatin regions and the rest of the nucleus. Representative chromatin density profiles for the individual experimental variants are shown in Figure 6A′–E′.

### 2.5. HU-Induced Changes in DNA Replication

**Figure 7:**
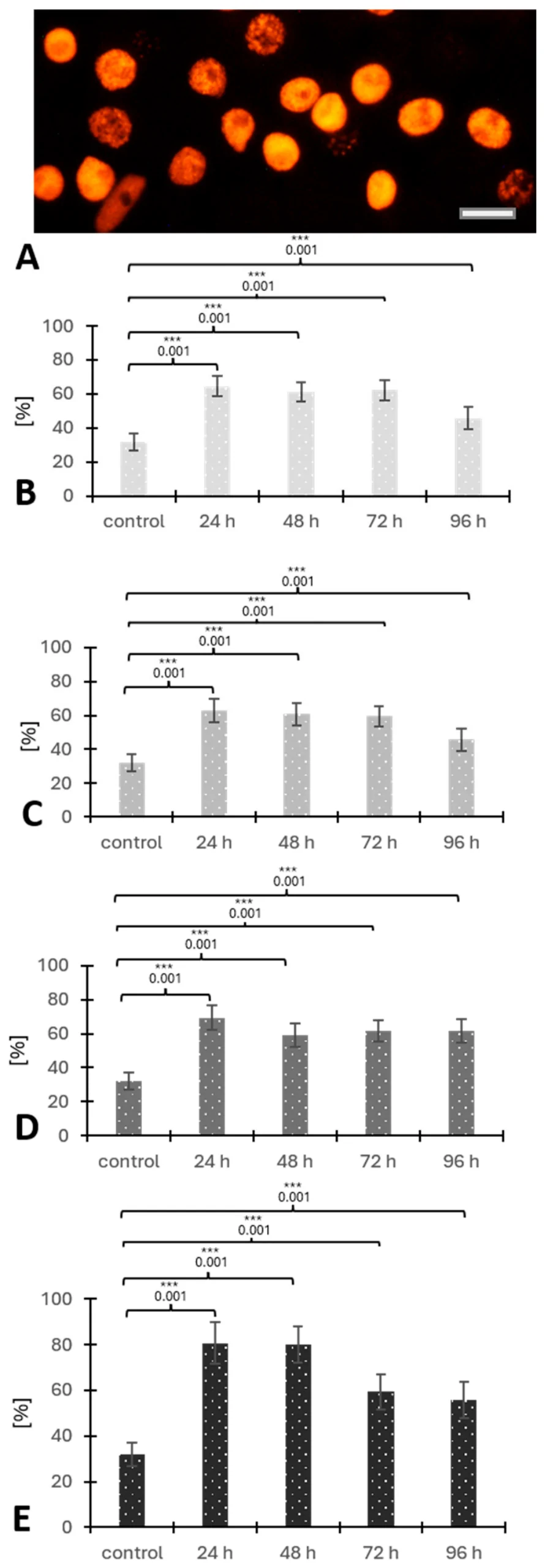
DNA replication in *A. cepa* root meristem cell populations evidenced by incorporation of EdU and copper(l)-catalyzed Alexa Fluor^®^555 azide-alkyne “click” reaction: (**A**) control. Scale bar = 20 µm. Fractions [(%) ± SD] of *A. cepa* root meristem cells, calculated by combining microfluorimetric quantitation of DNA contents in DAPI-stained cell nuclei and visual EdU fluorescence analysis: (**B**) 1 mM, (**C**) 2 mM (**D**) 3 mM and (**E**) 4 mM HU. Data are shown as mean (±SD; *n* = 3 biological replicates); approximately 500 nuclei were analyzed per treatment. When compared with the control, statistically significant changes in mean values (±SD) are marked by asterisk: *** *p* < 0.001 (Student’s *t*-test).

A detailed analysis of cell nuclei in meristematic onion cells revealed a clear effect of all tested HU concentrations on the process of DNA biosynthesis. Replicating nuclei were identified based on a combined microfluorometric analysis of DNA content in DAPI-stained nuclei and assessment of the EdU fluorescence signal (Figure 7A).

Incubations of onion root meristems with 1 and 2 mM HU, resulted in a similar increase in the number of replicating cell nuclei, compared with the data from control samples (Figure 7B,C). After 24, 48 and 72 h of treatment, the proportion of cells synthesizing DNA remained at a level of about 60%. At the final time point, however, a slight decrease in the number of fluorescently labelled nuclei was observed to a value of approximately 45% (Figure 7B,C).

During incubation of onion root meristems with 3 mM HU, the greatest increase in replicating cells was observed within the first 24 h. At the remaining time points (48, 72 and 96 h), the labelling index fluctuated at around 60% (Figure 7D). The highest HU concentration of 4 mM produced the greatest changes. Already after 24 and 48 h of incubation, the highest percentage of fluorescently labelled cells, approximately 80%, was recorded (Figure 7E). In contrast, at the last two time points, the number of replicating nuclei decreased to approximately 60% (Figure 7E).

The relatively high proportion of EdU-positive nuclei indicates that DNA synthesis persisted in a significant proportion of the cells treated with HU. However, this does not mean that replication proceeded at the normal rate or was completed. Therefore, these results reflect detectable DNA synthesis during the EdU pulse, rather than, directly, the progress of the RFs or the efficiency of replication.

### 2.6. Enhanced Levels of Hydrogen Peroxide (H_2_O_2_) and Superoxide Anion (O_2_∙^−^) Following Prolonged Exposure to HU

**Figure 8:**
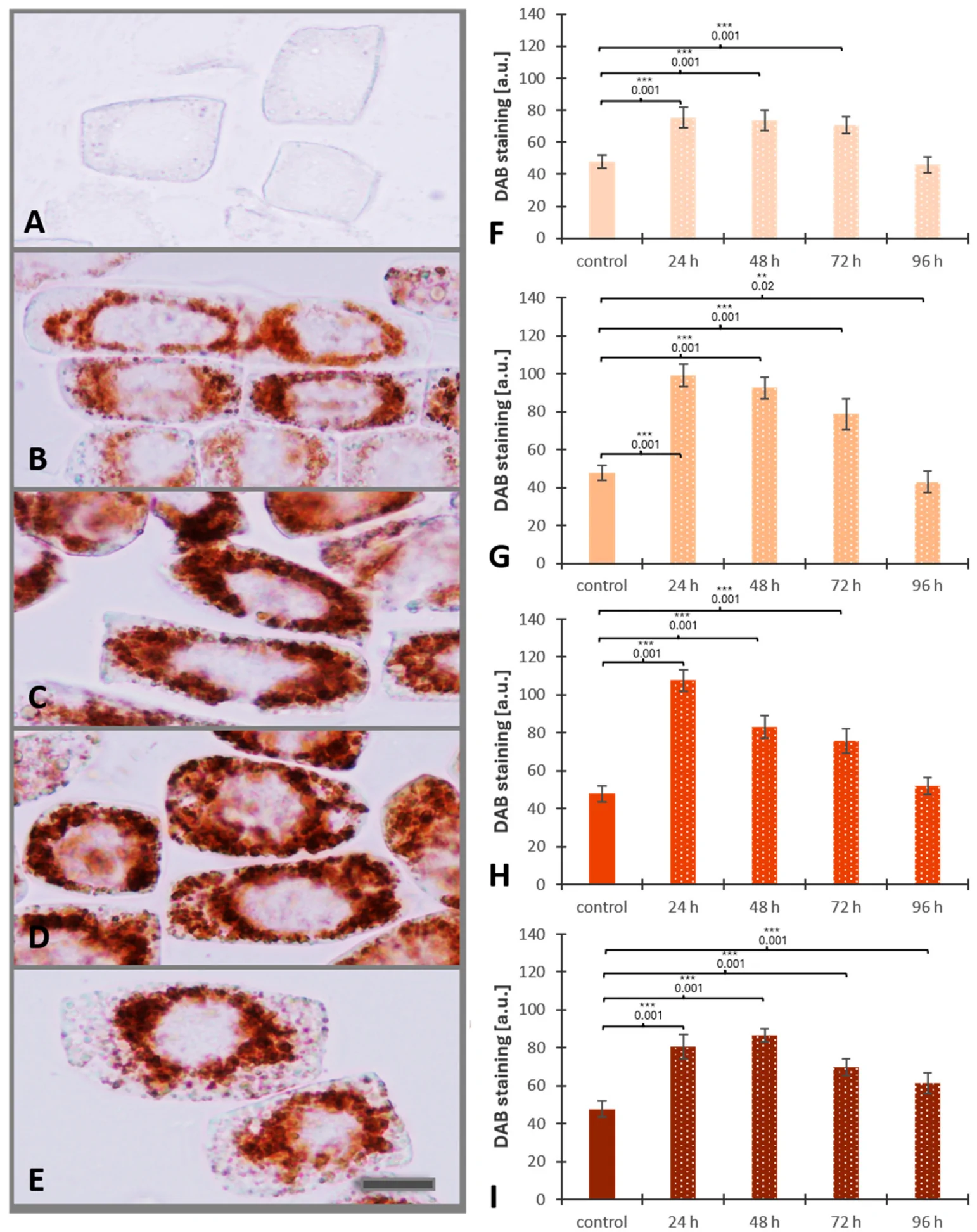
Generation of H_2_O_2_ in *A. cepa* meristem cells following prolonged treatment with 1, 2, 3, and 4 mM HU. (**A**) DAB-stained cells in the control cells, and after 24 h treatment with: (**B**) 1, (**C**) 2, (**D**) 3, and (**E**) 4 mM HU. Scale bar = 20 μm. (**F**,**I**) Microcolorimetric evaluation of the mean DAB staining intensities (arbitrary units (a.u.)) following induction in (**F**) 1, (**G**) 2, (**H**) 3, and (**I**) 4 mM HU. Data are shown as mean (±SD; *n* = 3 biological replicates); approximately 20 cells were analyzed per treatment. When compared with the control, statistically significant changes in mean values (±SD) are marked by asterisk: ** *p* <0.02, *** *p* < 0.001 (Student’s *t*-test).

In order to determine the levels of reactive oxygen species (ROS) induced by HU and to assess the harmful effects of 24, 48, 72 and 96 h exposures to 1, 2, 3 and 4 mM HU on *A. cepa* root meristem cells, two types of microcolorimetric analyses were employed, concerning concentrations of (1) H_2_O_2_ and (2) superoxide anion radical (O_2_∙^−^) (Figure 8).

H_2_O_2_ was detected both in the control onion root meristems (Figure 8A) and in HU-treated root samples (Figure 8B–E) using 3,3′-diaminobenzidine (DAB), which polymerizes into reddish-brown precipitates in the presence of peroxidase [26]. Microcolorimetric analysis showed that, as early as in the first day of exposure to HU, the intensity of H_2_O_2_-associated DAB staining, increased in all experimental variants, compared to the control group (Figure 8F–I). The highest value was recorded for a concentration of 3 mM HU (Figure 8H), while the lowest was observed for roots treated with 1 mM HU (Figure 8F). In the subsequent hours of incubation in the presence of the inhibitor, DAB staining intensity gradually decreased in all experimental systems, reaching values similar to those of the control, with the exception of the variant treated with 4 mM HU (Figure 8F–I).

**Figure 9:**
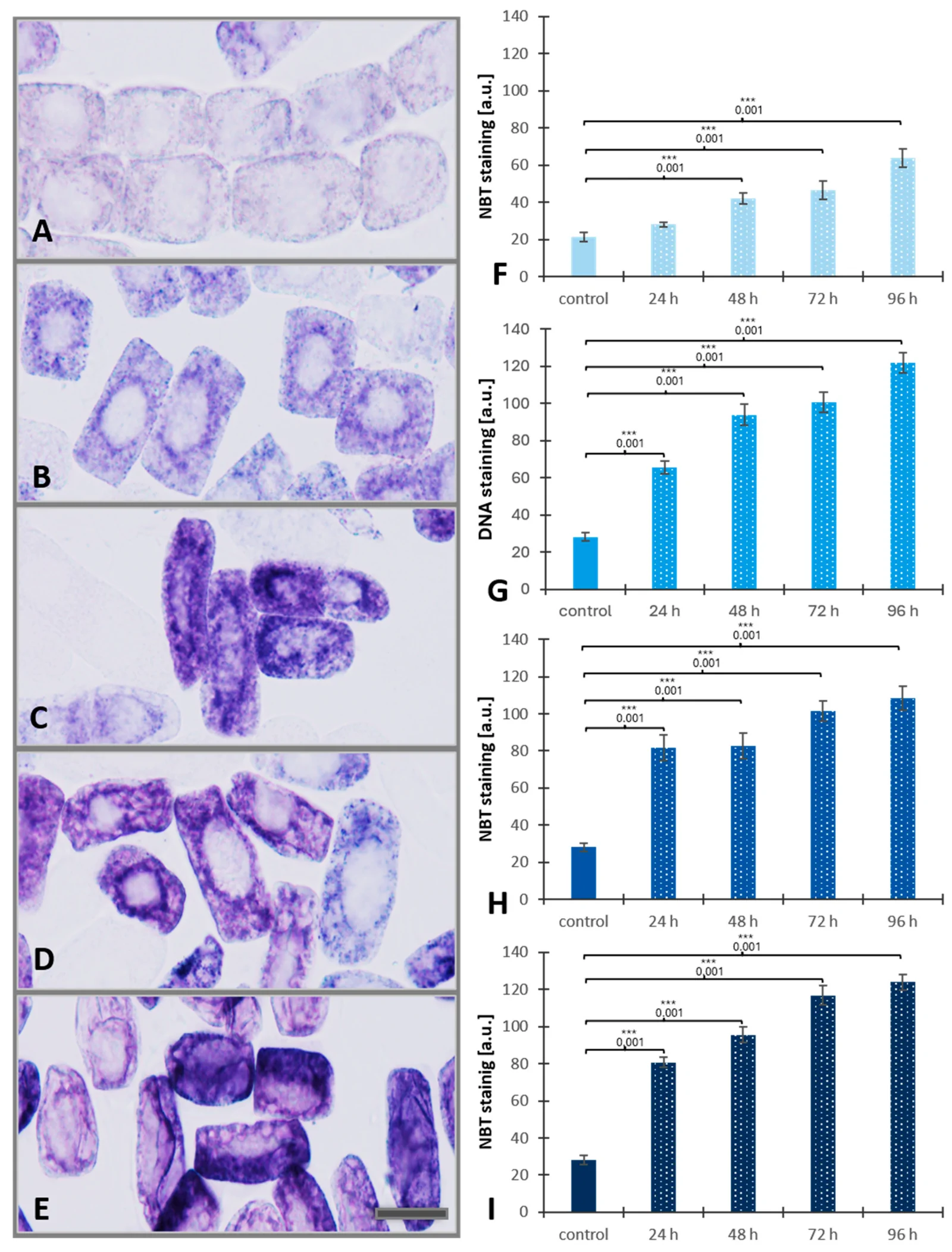
Localization of O_2_∙^−^ using NBT staining in root meristem cells in the control and following prolonged treatment with 1, 2, 3, and 4 mM HU. (**A**) NBT-stained cells in the control cells, and after 24 h treatment with: (**B**) 1, (**C**) 2, (**D**) 3, and (**E**) 4 mM HU. Scale bar = 20 μm. (**F**,**I**) Microcolorimetric evaluation of the mean NBT staining intensities [arbitrary units (a.u.)] following incubations in (**F**) 1, (**G**) 2, (**H**) 3, and (**I**) 4 mM HU. Data are shown as mean (±SD; *n* = 3 biological replicates); approximately 20 cells were analyzed per treatment. When compared with the control, statistically significant changes in mean values (±SD) are marked by asterisk: *** *p* < 0.001 (Student’s *t*-test).

The presence of the superoxide anion radical (O_2_∙^−^) was detected in accordance with the procedure described by Bielski et al. [27] using the blue-violet formazan formed from nitroblue tetrazolium chloride (NBT) (Figure 9A–E). Compared to the control group (Figure 9A), in which only small amounts of formazan deposits were observed, the intensity of O_2_∙^−^ staining in seedlings treated for 96 h with 1 mM HU reached a value almost three times higher (Figure 9F). Following treatment with 2, 3 and 4 mM HU, high levels of O_2_·^−^ were recorded after all incubation times (Figure 9G–I). On the fourth day of exposure to HU, the observed O_2_∙^−^ staining intensity was almost four times higher than that in the control group (Figure 9G–I).

### 2.7. Acetylation of Histone H3 on Lysine 56 (H3K56Ac)

**Figure 10:**
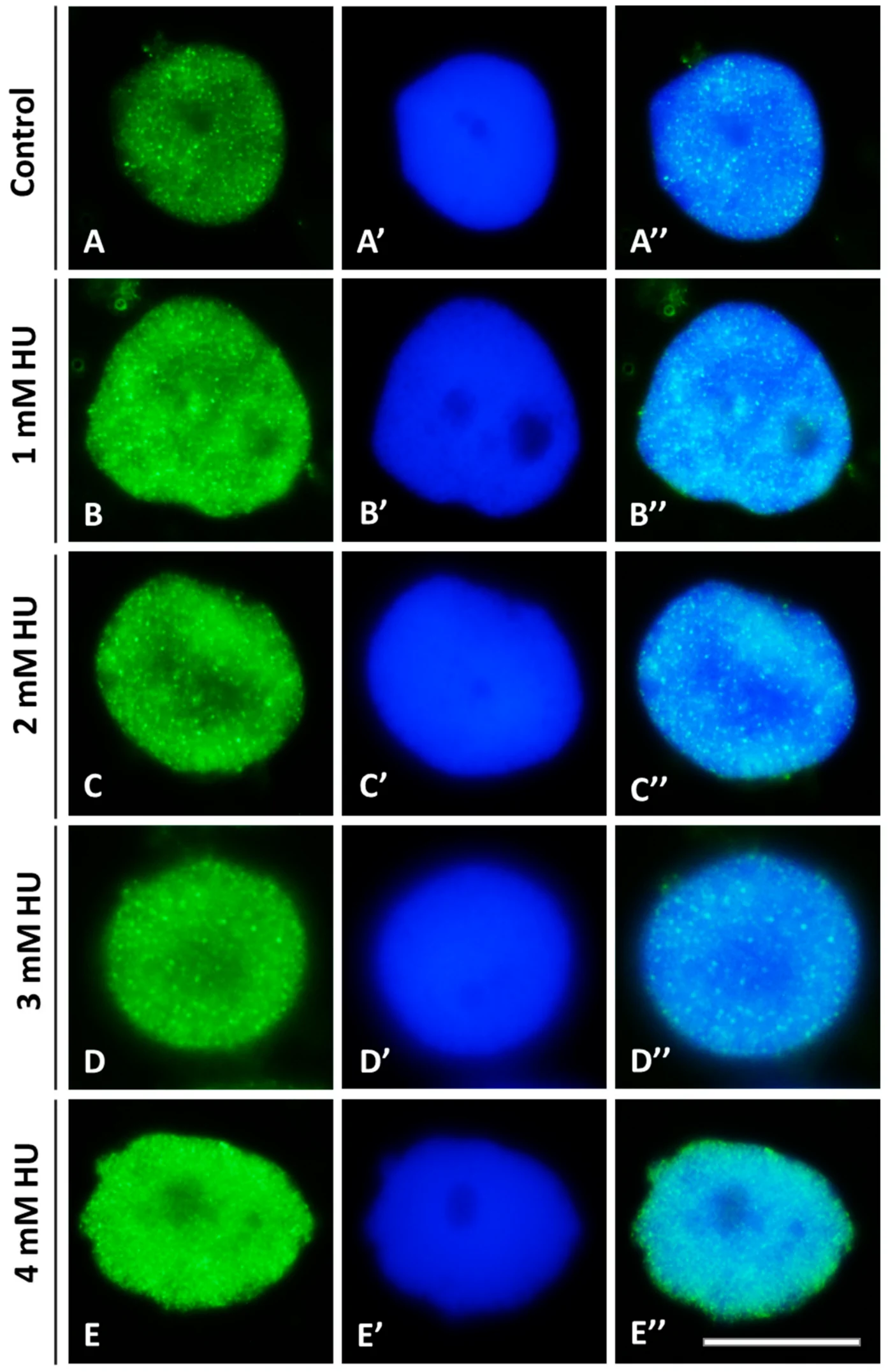
Immunofluorescence of H3K56Ac in cell nuclei in S-phase from: (**A**) control and after 24 h treatment with (**B**) 1, (**C**) 2, (**D**) 3 and (**E**) 4 mM HU. Nuclei stained with DAPI (**A′**–**E′**) and merged images (**A″**–**E″**). Scale bar = 10 μm.

Acetylation of histone H3 at lysine 56 (H3K56Ac) is an important epigenetic modification involved in the cellular response to DNA damage. Microscopic observations revealed that both the cells from the control group (Figure 10A,A′,A″) and those treated with 1, 2, 3, or 4 mM HU (Figure 10B,B′,B″–E,E′,E″) were characterized by the presence of a smaller or larger number of fluorescent foci (spots) of H3K56Ac located within the nuclear chromatin. Our previous studies on *Vicia faba* [21,28,29] concerning this modification involved calculating the average number of foci. Since the average number of H3K56Ac foci would be difficult to count in *A. cepa*, our present analyses covered the fluorescence intensity (FI) of the entire areas of cell nuclei (including the nucleoli) during the S-phase.

**Figure 11:**
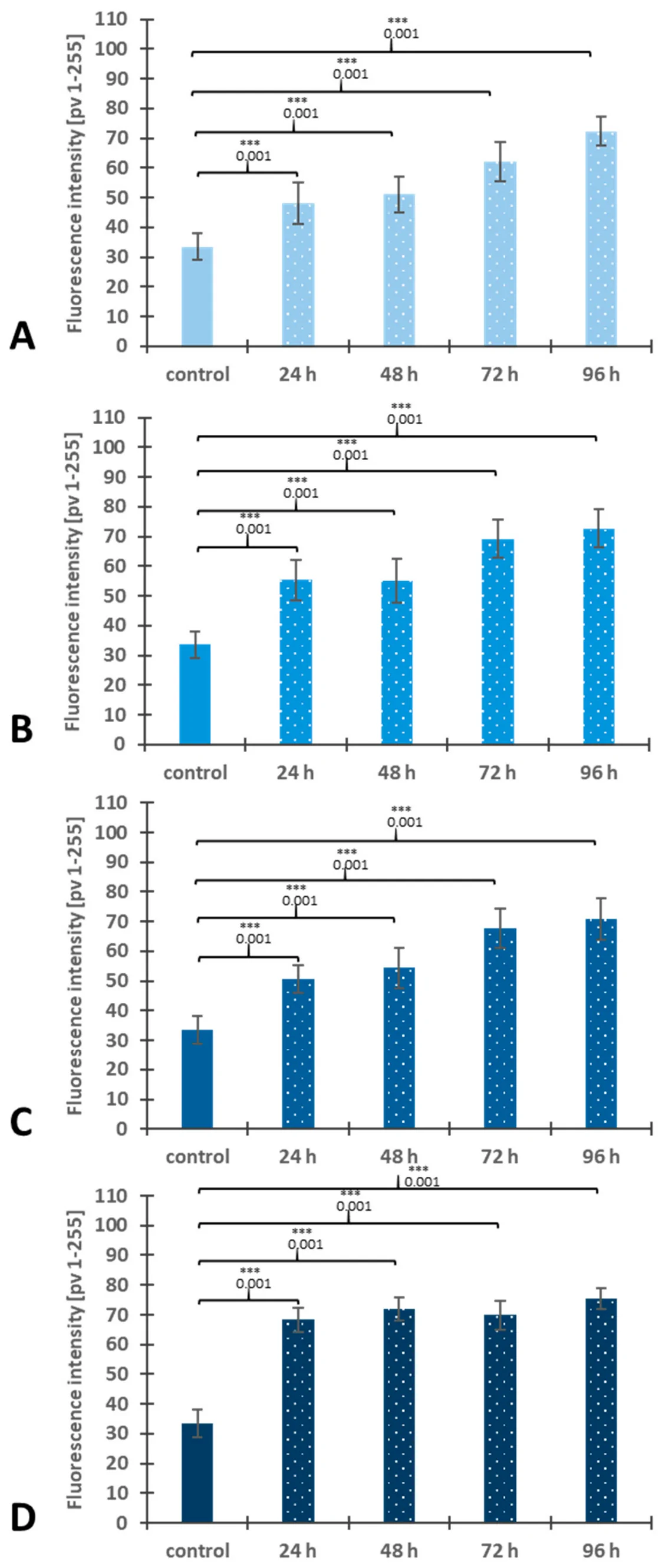
Mean fluorescence intensity of H3K56Ac in S-phase nuclei in the control (**A**–**D**) and following 24, 48, 72 and 96 h treatments with (**A**)1, (**B**) 2, (**C**) 3, and (**D**) 4 mM HU. Data are shown as mean (±SD; *n* = 3 biological replicates); approximately 20 nuclei were analyzed per treatment. When compared with the control, statistically significant changes in mean values (±SD) are marked by asterisk: *** *p* < 0.001 (Student’s *t*-test).

Compared to the control group, similar increases in the average fluorescence intensity of nuclei were observed after 24 and 48 h of incubation with 1, 2, and 3 mM HU (Figure 11A–C). The greatest increase in FI at both of these time points was observed after treatment with 4 mM HU (Figure 11D). Further increases in nuclear fluorescence occurred after 72 and 96 h of incubation with HU; the highest mean fluorescence intensity of nuclear chromatin was observed after 96 h of HU treatment, with these values being similar for all concentrations of the inhibitor (Figure 11A–D).

## 3. Discussion

Hydroxyurea (HU) is a commonly used DNA replication inhibitor that causes replication fork stalling (FRs). Its mechanism of action involves the selective inhibition of class I ribonucleotide reductase (RNR), an enzyme complex responsible for converting nucleotide diphosphates (NDPs) into deoxynucleotide diphosphates (dNDPs), which are essential for the production of deoxynucleotide triphosphates (dNTPs). RNR is a heterotetrameric enzyme, comprising a dimer of the large catalytic subunit (Rnr1 or its paralogue Rnr3) and a dimer of the small regulatory subunit (Rnr2 and/or Rnr4) [30,31,32]. HU inhibits RNR by reductively quenching the tyrosyl radical located at the di-iron (Fe^2+^/Fe^3+^) centre of the enzyme’s R2 subunit. HU acts as an electron donor, leading to the conversion of the tyrosyl radical into an inactive form, which results in the catalytic inactivation of the enzyme. Consequently, the pool of dNTPs is depleted, which prevents normal DNA synthesis and leads to the cessation of DNA polymerase activity [31,32]. It has also been shown that HU causes the accumulation of reactive oxygen species (ROS), such as H_2_O_2_. This phenomenon may be a consequence of chain radical reactions initiated by the hydroxylamine group [31,33]. ROS may exert teratogenic effects through direct DNA damage, and the level of DNA oxidation increases with HU administration [34,35].

Our previous work [16] revealed that prolonged incubation with low doses of HU (ranging from 0.50 to 0.75 mM) resulted in the production of large, elongated cells in the root meristems of *Allium cepa*. It was found that a relatively small number of mitotic divisions were observed throughout the treatment period, with a significant increase 72 h after the start of incubation, most of which exhibited clear signs of chromosomal aberrations characterized by premature chromosome condensation [PCC;16]. Our current studies, using various concentrations of HU—1, 2, 3 and 4 mM—also resulted in larger nuclei with distinct chromocentres. Analyses of mitotic activity following HU treatment also yielded interesting results. HU affected mitotic activity in a concentration- and time-dependent manner. We demonstrated that, throughout the entire incubation period, a lower number of mitotic divisions was observed in the presence of 1 and 2 mM HU. The exception was a significant increase in the number of mitoses recorded after 48 h of incubation with 1 mM HU and after 72 h of incubation with 2 mM HU, which suggests that the effect of HU on mitotic activity was not consistent across all experimental conditions. The most pronounced decrease in the mitotic index was observed following treatment with the highest HU concentration (4 mM). At all analyzed time points, the relative number of mitotic cells remained at a very low level, ranging from 0.2% to 0.6%. Furthermore, we observed that the highest proportion of chromosomal aberrations (12–16%) occurred following incubation of the roots in 1 mM HU. It is interesting to note that higher concentrations of HU did not lead to an increase in the frequency of chromosomal aberrations, which may have been due to a lower number of cells in mitosis. Therefore, based on our previous studies [16], we can conclude that while the marked decrease in the frequency of cells in mitosis reflects a secondary effect of DNA synthesis inhibition, the small proportion of cells entering the PCC should be interpreted as a consequence of mechanisms that enable the bypassing of the DNA RS response pathway, also referred to as the S-phase checkpoint [36]. These mechanisms allow cells to enter premature mitosis despite incomplete replication of nuclear DNA [37,38,39].

The persistence of a relatively high proportion of EdU-positive nuclei during treatment of meristematic root cells of *A. cepa* HU does not necessarily rule out the occurrence of RS. The incorporation of EdU indicates that detectable DNA synthesis took place during the labelling period, but does not allow to estimate the rate, continuity or successful completion of DNA synthesis. Under conditions of dNTP depletion, DNA synthesis may continue despite the fact that the course of replication is disrupted. The reduced mitotic activity and increased frequency of chromosomal aberrations observed in this study are consistent with disruptions of the cell cycle and in the genomic stability. However, as the speed of the replication forks, as well as their arrest and collapse, were not measured directly, the present results do not allow for the quantitative determination of these processes.

Our current research indicates that prolonged incubation of 4-day-old primary roots of *A. cepa* with a 1 mM HU solution can lead to the formation of nuclear chromatin structures of both interphase and mitotic nature (I-M cells). These structures were not observed following treatment with other concentrations of HU. A characteristic feature of these structures is partial chromatin condensation—one part remains decondensed, which is typical of interphase, while the other undergoes condensation, characteristic of prophase. This phenomenon was discovered several years earlier in *A. cepa* cells following a 3-day exposure to 0.75 mM HU [16,17,18,19,20] and to 0.75 mM 5-aminouracil (5-AU) [40], as well as in *V. faba* cells [21]. It has been shown that prolonged RS induced by HU can lead to increased levels of cyclin B-like proteins. It is thought that it is the gradient of these proteins, formed along the cell axis, that is responsible for the formation of biphasic nuclear structures, containing both interphase and mitotic chromatin domains [16]. The low frequency of I-M cells represents a limitation when interpreting the quantitative relationship between HU concentration and the occurrence of this nuclear phenotype. Accordingly, the observed pattern should be considered indicative of altered chromatin organization under replication stress rather than definitive evidence of S-phase checkpoint bypass. Several alternative explanations may account for the concentration-dependent pattern, including differences in the severity of replication stress, the extent of cell-cycle progression impairment, and the capacity of meristematic cells to adapt to prolonged HU exposure. In addition, the frequency of I-M cells may reflect differences in cellular sensitivity and the transient nature of this chromatin configuration. As checkpoint activity and the temporal progression of individual I-M cells were not directly assessed, all the above possibilities remain hypothetical.

According to the study by Wilhelm et al. [41], oxidative stress (OS) and RS are interrelated. The interaction between these processes is one of the key sources of genomic instability. OS can influence DNA replication via three main, parallel mechanisms. Firstly, OS induces DNA damage leading to RS, as certain types of oxidized bases inhibit the movement of DNA polymerases. Furthermore, DSBs induced by OS can lead to the collapse of replication forks. Secondly, oxidative modifications of proteins involved in the replication process can disrupt the initiation of DNA biosynthesis. Thirdly, changes in the cell’s redox state significantly affect the dNTP pool, which in turn may disrupt the normal course of DNA replication [41]. Analyses based on the DAB and NBT methods revealed treatment-related changes in staining intensity, consistent with specific signals associated, respectively, with H_2_O_2_ and O_2_•^−^However, colorimetric assays do not allow for the direct measurement of absolute ROS concentrations. Consequently, the observed differences should be interpreted as relative changes in colour intensity, rather than as definitive quantitative measurements of intracellular levels of H_2_O_2_ and O_2_•^−^. In our study, microcolorimetric analyses showed that, as early as the first day of exposure to various concentrations of HU, the intracellular H_2_O_2_ level was higher than in the control group. The highest value was recorded for a concentration of 3 mM HU, while the lowest was for 1 mM HU. Interestingly, within a few hours of administering the inhibitor, the DAB staining intensity associated with H_2_O_2_ gradually decreased in all experimental systems, reaching values similar to those in the control group, with the exception of the variant treated with 4 mM HU. In the case of the O_2_·^−^ analysis, a gradual increase in O_2_·^−^ production was observed in root meristem samples treated with 1 mM HU, reaching a level almost three times higher than that in the control group after 96 h of exposure to HU. Following the addition of higher concentrations of HU (2, 3 and 4 mM), an increase in the intensity of the NBT colouration associated with O_2_•^−^ was observed at all the incubation times analyzed. The increase in H_2_O_2_ after 24 h, followed by a decrease, may result from the transient activation of ROS-generating processes, then followed by an increase in their removal or a reduction in their production. H_2_O_2_ can be eliminated, among other factors, by catalase, peroxidases and peroxidoxin systems. Conversely, a sustained increase in O_2_•^−^may indicate that its production outweighs its removal, or that sources of superoxide anion-radical remain active. The action of superoxide dismutase converts O_2_•^−^ into H_2_O_2_, which is why the dynamics of the two forms of ROS need not be parallel. Accumulated ROS may therefore contribute to teratogenic effects by directly damaging DNA, and the degree of DNA oxidation increases during exposure to HU [35,42,43]. A growing body of evidence suggests that ROS and the cellular damage pathways they induce account for a significant proportion, and perhaps even the majority, of HU’s acute cytotoxicity [31,32,33].

In this study, we demonstrated that both control cells and those treated with 1, 2, 3 and 4 mM HU were characterized by the presence of a smaller or larger number of fluorescent H3K56Ac foci localized within the nuclear chromatin. A similar increase in the mean fluorescence intensity of H3K56Ac was observed following 24- and 48 h incubation with 1, 2 and 3 mM HU, with the highest intensity recorded after treatment with 4 mM HU. Subsequent hours of incubation with the inhibitor (72 and 96 h) resulted in a further increase in nuclear fluorescence. Since H3K56 acetylation is also associated with newly synthesized histone H3 and the formation of nucleosomes linked to replication. In yeast, this process promotes the interaction of histones H3–H4 with factors responsible for chromatin structuralization, including CAF-1 and Rtt106, which facilitate nucleosome formation on newly replicated DNA [22]. It can therefore be assumed that the increase in H3K56Ac levels observed following HU administration may reflect changes in chromatin organization and maturation associated with replication.

This study has several limitations which should be taken into account when interpreting the results. Firstly, signals associated with the presence of reactive oxygen species were assessed on the basis of DAB and NBT histochemical staining, which provide indirect, semi-quantitative indicators associated with H_2_O_2_ and the superoxide anion (O_2_•^−^), respectively, but do not allow for the direct determination of their intracellular concentrations. Similarly, changes in the intensity of H3K56Ac immunofluorescence reflect changes in the signal associated with the histone modification under investigation, but do not constitute direct evidence of the extent of DNA damage or the efficacy of DNA repair processes. Secondly, the use of a limited number of biological replicates may reduce the statistical power of the analysis and limit the generalizability of the results obtained. Furthermore, the study was conducted exclusively on the *Allium cepa* model, which does not allow us to determine whether the observed responses to HU-induced replicative stress also occur in other plant species. Verification of the presented observations requires further research involving independent methods for the quantitative assessment of ROS, a greater number of biological replicates and additional plant models.

## 4. Materials and Methods

### 4.1. Plant Material

Onion seeds (*Allium cepa*) were surface-sterilized with 70% (*v*/*v*) ethanol for 3 min and 10% (*v*/*v*) bleach with 0.01% (*v*/*v*) Triton X-100 (Merck Life Science, Poznan, Poland) for 5 min. After rinsing the seeds three times with sterile water, they were sown in Petri dishes on moist tissue of filter paper. The cultures were maintained at 24 °C. After 96 h, seedlings with roots approximately 2 cm long were placed in subsequent dishes containing distilled water (control) and 1, 2, 3 and 4 mM hydroxyurea (HU; Merck Life Science, Poznan, Poland) solution. Incubation was carried out for 24, 48, 72 and 96 h in the dark.

### 4.2. Feulgen Staining

Cut root meristems were fixed in Carnoy’s mixture (absolute ethanol and glacial acetic acid; 3:1, *v*/*v*) for 60 min. After rinsing with ethanol (Merck Life Science, Poznan, Poland), the material was subjected to a 1 h hydrolysis in 4 M HCl (Merck Life Science, Poznan, Poland). Then, the roots were stained with Schiff’s reagent (Merck Life Science, Poznan, Poland). After rinsing with distilled water, meristems were squashed on microscope slides (Merck Life Science, Poznan, Poland) in a drop of 45% acetic acid. After freezing on dry ice (10 min), the slides were rinsed with 70% (*v*/*v*) ethanol. After drying, the slides were embedded in Canada balsam (Merck Life Science, Poznan, Poland).

### 4.3. Hematoxylin Staining of Semi-Thin Sections

Meristematic zones from control *A. cepa* roots and those treated for 72 h with 1 mM HU were fixed in a solution of 1% cacodylate-buffered (pH 7.3) 3% glutaraldehyde (Merck Life Science, Poznan, Poland), dehydrated in a series of alcohol (1, 30, 50, 70%) and embedded in the Epon 812 and Spurr mixture medium (Polysciences, Poznan, Poland). Semi-thin sections were cut using Ultramicrotome (Ultracut E, Reichert-Jung, Vienna, Austria), and stained with haematoxylin (Merck Life Science, Poznan, Poland). An E600 epifluorescence microscope (Nikon, Tokyo, Japan) was used to photograph semi-thin sections.

### 4.4. Identification of Replicating Nuclei with 5-Ethynyl-2′-deoxyuridine (EdU)

To determine the percentage of cells exhibiting detectable DNA synthesis, onion root meristems grown in water and HU solutions were incubated with 10 μM 5-ethynyl-2′-deoxyuridine (EdU; Thermo Fisher Scientific, Warsaw, Poland). EdU was administered for 30 min after 24, 48, 72 and 96 h HU treatments. The results were interpreted as a point estimate of DNA synthesis activity, rather than as a direct measure of the proportion of the S-phase over the entire period of HU exposure. The dissected meristems were fixed in 4% phosphate-buffered saline (PBS)–buffered paraformaldehyde (4 °C; 20 min; pH 7.4; Merck Life Science, Poznan, Poland) and macerated with 2.5% citrate-buffered pectinase (pH 5.0; 30 min; Merck Life Science, Poznan, Poland). Meristems were squashed on PolysineTM slides (Merck Life Science, Poznan, Poland). Replicating cells were visualized using the Click-iT DNA Alexa Fluor^®^555 Imaging Kit (Thermo Fisher Scientific, Warsaw, Poland), according to the supplier’s manuals. Finally, DNA was stained for 5 min with 15 μM 4′,6-diamidino-2-phenylindole (DAPI; Merck Life Science, Poznan, Poland). The slides were mounted in a mixture of PBS/glycerol/diazabicyclo(2.2.2)octane (DABCO, 2.3%; Merck Life Science, Poznan, Poland).

### 4.5. Detection of H_2_O_2_ Using DAB Staining and O_2_ ^∙−^ Using Nitroblue Tetrazolium (NBT) Test

In the control cells and those treated with 1, 2, 3 and 4 mM HU, H_2_O_2_ was detected using the 3,3-diaminobenzidine tetrachloride (DAB-HCl; Merck Life Science, Poznań, Poland) staining method, in accordance with the procedure described by Thordal-Christensen et al. [26]. DAB reacts with H_2_O_2_ in the presence of peroxidase forming a brown polymerized product. The seedlings were incubated for 3 h in 1 mg mL-1 DAB-HCl in TRIS buffer (10 mM Tris, 10 mM EDTA-2Na, 100 mM NaCl; pH 7.5) and then fixed in 3.7% PBS-buffered paraformaldehyde. After 15 min, the seedlings were washed with PBS and macerated in 2.5% citric acid-buffered pectinase solution (pH 5.0; 37 °C, 20 min). Root meristems were squashed onto slides and mounted in a mixture of glycerol and PBS (9:1; *v*/*v*). The appearance of a brown colour indicated the presence of H_2_O_2_.

Superoxide anion radical (O_2_∙^−^) was visualized by reducing nitro blue tetrazolium (NBT; Merck Life Science, Poznań, Poland) to coloured formazan precipitates according to Bielski et al. [27]. Seedlings grown in water and treated with HU were immersed for 10 min in a solution of 3 mM NBT in 10 mM Tris-HCl buffer (pH 7.4) at 24 °C. The procedure’s remaining steps were similar to those for the DAB assay.

### 4.6. Immunocytochemical Detection of Histone H3 Acetylation on Lysine 56 (H3K56Ac)

Meristems dissected from the control roots and from those treated with 1, 2, 3 and 4 mM HU were fixed for 15 min (4 °C) in PBS-buffered 4% paraformaldehyde. Then, cell nuclei were isolated by crushing the root meristems between two basal slides according to Żabka et al. [28]. After thorough rinsing with PBS, slides were pretreated for 50 min with blocking buffer containing 8% bovine serum albumin (BSA; Merck Life Science, Poznań, Poland) and 0.3% Triton X-100 (Sigma-Aldrich, Poznań, Poland) diluted in PBS in a wet chamber at 24 °C. The slides were then incubated with a rabbit monoclonal antihistone H3K56Ac antibody (catalogue number 326692, Abcam, Cambridgeshire, UK) dissolved in PBS containing 1% BSA (Merck Life Science, Poznan, Poland) at a dilution of 1:200. Incubations were conducted in a humidified atmosphere at 4 °C for overnight. Then, slides were washed with PBS and incubated for 2 h (24 °C) with secondary goat anti-rabbit immunoglobulin (IgG) conjugated to Alexa Fluor^®^488 antibody in PBS (catalogue number 4412, 1:400; Cell Signaling, Leiden, The Netherlands). A negative control was processed in parallel by omitting the primary antibody while retaining the remaining staining steps, including incubation with the secondary antibody. This control was used to assess nonspecific fluorescence associated with the secondary antibody and the staining procedure. The specificity of the antibody has been confirmed by validation data provided by the manufacturer for the specified applications, as described in the relevant product data sheets.

Cell nuclei were counterstained with DAPI (Merck Life Science, Poznan, Poland). The slides were mounted in a mixture of PBS/glycerol/DABCO (2.3% diazabicyclo(2.2.2)octane; Merck Life Science, Poznan, Poland).

### 4.7. Observations and Microscope Analyses

Observations of cells stained with EdU and with antibodies against histone modification H3K56Ac were made under a Nikon Eclipse E600 fluorescence microscope (Nikon, Tokyo, Japan) equipped with blue light (B2) filter (λ = 465–496 nm) for Alexa Fluor^®^488, green light (G2 filter) (λ = 540/25 nm) for Alexa Fluor^®^555 and UVB light (U2) filter (λ = 340–380 nm) for DAPI. All fluorescence images were recorded at the same integration time using a DS-Fi1 CCD camera and Nikon Eclipse E600 microscope (Tokyo, Japan). The images were selected for analysis on the basis of signal clarity and the visibility of cell nuclei. Nuclear DNA fluorescence measurements and quantitative analyses were obtained after converting colour images to grayscale, and the results were expressed in arbitrary units as mean pixel value (pv) spanning the range from 0 (dark) to 255 (white). A Nikon Eclipse E600 microscope was used to photograph the cell nuclei stained using the Feulgen method.

In order to obtain the percentage of mitotic cells (mitotic index) and aberrant M-phase cells (aberration index) after 24, 48, 72 and 96 h of incubation in 1, 2, 3, and 4 mM HU, 50 cells (stained using the Feulgen method) from each of the four root tips were analyzed. Three biological replicates were performed. Quantitative evaluation of chromosomal aberrations was based on the percentage of mitotic cells exhibiting structural abnormalities of chromosomes in relation to the total number of mitotic cells analyzed for each sample.

In order to distinguish cells in different phases of the cell cycle (the G1, S and G2 phases) a cytophotometric measurement of DNA content in the cell nucleus was carried out. The analysis was carried out using a Jenamed 2 microscope (Carl Zeiss, Jena, Germany) in conjunction with the Cytophotometer v1.2 software (Forel, Łódź, Poland). The analysis was carried out by combining microfluorometric assessment of DNA content in DAPI-stained cell nuclei with the identification of nuclei showing EdU incorporation. The EdU labelling index was calculated as the percentage of EdU-positive nuclei in relation to the total number of cell nuclei analyzed for each experimental condition. This index reflects the percentage of nuclei in which detectable DNA synthesis occurs during the 30 min labelling period and does not directly measure the total S-phase fraction over the entire period of HU exposure.

A quantitative analysis of H3K56Ac immunofluorescence was carried out using three independent biological replicates. Approximately 20 cell nuclei were analyzed under each experimental condition. Cell nuclei assigned to the S-phase were identified using cytometric measurement of DNA content (DNA content analysis was based on DAPI staining). Only nuclei with clearly distinguishable boundaries, allowing for reliable assessment of fluorescence, were included. The selection of nuclei was based on image quality and cell cycle identification criteria, rather than on the observed intensity of the H3K56Ac signal. Background fluorescence, calculated as a mean of five fluorescence measurements around each cell nucleus was assessed and subtracted from each region of interest within the nucleus. Image acquisition settings were kept constant for all samples under comparison.

DAB- and NBT-stained meristem cells were photographed under a Nikon Eclipse E600 miscroscope. Colour images were converted to a grey scale and expressed in arbitrary units as mean pixel value (pv), spanning the range from 0 (dark) to 255 (white). The ImageJ software ver. 1.54p (National Institutes of Health, Bethesda, MD, USA) program was used to analyze intensities of DAB and NBT staining. As DAB and NBT staining provide a colorimetric readout rather than a direct measurement of ROS concentration, the results were interpreted as relative changes in staining intensity associated with the detection of H_2_O_2_ and O_2_•^−^, respectively. Because extremely low readouts for background staining were recorded in each of these experimental series, it was decided that correcting the data in both cases was unnecessary.

Following background correction, the mean grayscale value was measured in each cell region of interest and in the adjacent unstained background region using ImageJ software version 1.54p. Because DAB and NBT reaction products appeared as dark deposits, the relative staining signal was calculated as the difference between the mean grayscale value of the background and that of the cell.

Chromatin density profiles were generated for all experimental series using ImageJ software ver. 1.54p, enabling an assessment of changes in nuclear chromatin organization under the influence of HU.

All fluorescence assays were repeated at least three times.

### 4.8. Statistical Analyses

The Student’s *t*-test was used to determine the difference between the averages of two independent groups. The data obtained from all experiments were expressed as mean values ± standard deviation of the mean (±SD).

## 5. Conclusions

Prolonged (24, 48, 72 and 96 h) exposure of *A. cepa* root meristems to various concentrations of HU (1, 2, 3 and 4 mM) induced RS, as indicated by changes in EdU labelling patterns, reduced mitotic activity, chromosomal aberrations and altered chromatin organization. Although these results are consistent with disturbances in DNA replication and cell cycle progression, the present study does not allow for a direct determination of the dynamics of RFs or the extent of their arrest and collapse.

It has been shown that treatment with HU was associated with changes in colour intensity in the DAB and NBT assays, indicating changes in signals associated with H_2_O_2_ and the superoxide anion radical (O_2_•^−^), respectively. This was accompanied by chromosomal aberrations and the occurrence of premature chromosome condensation, indicating the possibility of S-phase checkpoint bypass and premature entry of cells into mitosis. Furthermore, at a concentration of 1 mM HU, characteristic nuclear structures combining features of interphase and mitotic chromatin were observed, which may indicate a disruption in the normal regulation of processes related to chromatin condensation and the cell cycle.

Another significant finding was the increase in the level of histone H3K56Ac acetylation during prolonged exposure to HU. This phenomenon likely reflects the activation of DNA damage response mechanisms and repair processes and suggests that changes in chromatin organization play a role in the cellular response to RS.

In summary, the results obtained indicate that HU induces a complex response in *A. cepa* cells, involving both RS and OS, DNA damage, cell cycle control abnormalities, chromosomal aberrations, and changes in chromatin structure and modifications. The interaction between these processes may be a key mechanism leading to genomic instability and impaired cell proliferation in cells exposed to prolonged HU treatment.

## Data Availability

The original contributions presented in this study are included in the article. Further inquiries can be directed to the corresponding author.
